# Impact of routine nasogastric decompression *versus* no nasogastric decompression after pancreaticoduodenectomy on perioperative outcomes: meta-analysis

**DOI:** 10.1093/bjsopen/zrab111

**Published:** 2021-12-21

**Authors:** Khaled Ammar, Chris Varghese, Thejasvin K, Viswakumar Prabakaran, Stuart Robinson, Samir Pathak, Bobby V M Dasari, Sanjay Pandanaboyana

**Affiliations:** 1 Department of Hepatobiliary, Pancreatic and Transplant Surgery, Department of Surgery, Freeman Hospital, Newcastle upon Tyne, UK; 2 Department of Hepato-Pancreato-Biliary Surgery, National Liver Institute, Menoufia University, Shebin El Kom, Egypt; 3 Department of Surgery, Faculty of Medical and Health Sciences, University of Auckland, Auckland, New Zealand; 4 Department of Surgery, Faculty of Medical Sciences, Newcastle University, Newcastle upon Tyne, UK; 5 Department of Hepatobiliary and Pancreatic Surgery, St James Hospital, Leeds, UK; 6 Department of Hepatobiliary, Pancreatic and Transplant Surgery, Queen Elizabeth Hospital, Birmingham, UK; 7 Population Health Sciences Institute, Newcastle University, Newcastle upon Tyne, UK

## Abstract

**Background:**

Consensus on the use of nasogastric decompression (NGD) after pancreaticoduodenectomy (PD) is lacking. This meta-analysis reviewed current evidence on the impact of routine NGD *versus* no NGD after PD on perioperative outcomes.

**Methods:**

PubMed, Medline, Scopus, Embase and Cochrane databases were searched for studies reporting on the role of NGD after PD on perioperative outcomes. Data up to January 2021were retrieved and analysed.

**Results:**

Eight studies were included, with a total of 1301 patients enrolled, of whom 668 had routine NGD. Routine NGD was associated with a higher incidence of overall delayed gastric emptying (DGE) (odds ratio (OR) 2.51, 95 per cent c.i. 1.12 to 5.63, *I*^2^ = 83 per cent; *P* = 0.03) and clinically relevant DGE (OR 3.64, 95 per cent c.i. 1.83 to 7.25, *I*^2^ = 54 per cent; *P* < 0.01), a higher rate of Clavien–Dindo grade II or higher complications (OR 3.12, 95 per cent c.i. 1.05 to 9.28, *I*^2^ = 88 per cent; *P* = 0.04) and increased length of hospital stay (mean difference 2.67, 95 per cent c.i. 0.60 to 4.75, *I*^2^ = 97 per cent; *P* = 0.02). There were no significant differences in overall complications (OR 1.07, 95 per cent c.i. 0.79 to 1.46, *I*^2^ = 0 per cent; *P* = 0.66) or postoperative pancreatic fistula (OR 1.21, 95 per cent c.i. 0.86 to 1.72, *I*^2^ = 0 per cent; *P* = 0.28) between patients with or those without routine NGD.

**Conclusion:**

Routine NGD was associated with increased rates of DGE, major complications and longer length of stay after PD.

## Teaser

There is no consensus regarding the role of nasogastric decompression after pancreaticoduodenectomy. This is a meta-analysis of current evidence showing that nasogastric decompression can be associated with higher rates of complications. However, further RCTs are needed, as there was only one RCT available for this meta-analysis.

## Introduction

Pancreaticoduodenectomy (PD) is the only curative treatment for periampullary, pancreatic, biliary tract and duodenal tumours. PD is associated with a high postoperative morbidity rate of between 30 and 50 per cent, despite major progress in operative techniques and perioperative care^1^^,^^2^. Postoperative morbidity, in turn, influences the quality of life and particularly oncologic outcomes due to delays in receiving adjuvant chemotherapy^3^.

Postoperative management after PD often includes placement of a nasogastric (NG) tube for gastric decompression. NG tubes are traditionally placed with a view to divert gastric juices and manage postoperative ileus and delayed gastric emptying (DGE). It is also commonly perceived that routine nasogastric decompression (NGD) after major abdominal surgery accelerates gastrointestinal functional recovery and reduces anastomotic leaks, gastric stasis, nausea and vomiting^4^^,^^5^. However, recent evidence suggests NGD may result in a delayed return of bowel function, higher pulmonary complication rates and a longer hospital stay^6–8^. It is generally agreed that routine NGD should no longer be used after liver, oesophageal, gastric or colorectal surgeries^6^^,^^7^^,^^9–12^. With regard to pancreatic surgery, the Enhanced Recovery After Surgery (ERAS) guidelines recommend against the routine use of NGD after PD^13^^,^^14^. A recent single-centre RCT also found that there is no significant difference in the occurrence of Clavien–Dindo grade II or higher complications, DGE or length of hospital stay following routine NGD after PD^15^. There is a lack of consensus around the necessity of routine NG tube placement after PD, with previous systematic reviews limited to only retrospective and non-randomized studies^16^.

Therefore, this meta-analysis aims to review the current evidence on the impact of routine NGD following PD on perioperative outcomes in light of a recently published RCT^15^.

## Methods

The study was prospectively registered on PROSPERO (CRD42021230650).

### Data sources and searches

This study was reported according to PRISMA criteria^17^. PubMed, Medline, Scopus, Embase and Cochrane databases were searched for studies reporting the role of NG tube decompression after PD and perioperative outcomes up to January 2021. The following query terms were employed: the combined results of ‘pancreaticoduodenectomy’ OR ‘Whipple’ OR ‘pancreatic surgery’ AND the combined results of ‘gastric decompression’ OR ‘nasogastric decompression’ or ‘nasogastric tube’ AND the combined results of ‘trials’ OR ‘randomised’ OR ‘randomized controlled trial’. There were no date or language restrictions. Two authors (K. A., V. P.) undertook the search independently and when there was a disagreement, the senior author was consulted (S. P.).

### Study selection

Single and multicentre retrospective or prospective cohort studies and randomised controlled studies investigating the role of NG tube insertion after PD were included. Review articles, case reports, conference abstracts, letters and non-English articles were excluded. Studies comparing NGD to gastrostomy decompression were also excluded.

## Definitions

DGE was defined and graded into three grades A, B and C, based on the International Study Group of Pancreas Surgery (ISGPS) classification^18^. Grade A DGE was diagnosed if patients required an NG tube between postoperative days (PODs) 4 and 7 (including reinsertion after initial removal) or in those who failed to tolerate a solid diet by POD 7 but could tolerate a solid diet before POD 14. Grade B DGE was diagnosed in patients who required an NG tube from POD 8 to 14 (including reinsertion after initial removal) or in those not tolerating solid oral intake by POD 14 but could tolerate solid oral intake by POD 21. Grade C DGE was considered if patients required an NG tube after POD 14 (including reinsertion after initial removal) or in those who could not maintain solid oral intake by POD 21^18^. Grade B and C DGE was considered clinically relevant DGE (CR-DGE).

POPF was defined and graded A, B or C according to the International Study Group on Pancreatic Fistula (ISGPF) definition in 2005^1^. The definition and grading of POPF were updated in 2016, according to the ISGPS classification^19^. Studies using either of these definitions were included in this review.

## Outcome measures

The primary outcome measure was the effect of routine NGD *versus* no NGD on DGE and CR-DGE rate. The secondary outcome measures were overall complications, Clavien–Dindo grade 0–I and II or higher complications, POPF, POPF grades B/C, bile leak, time to tolerate oral fluid and solid intake, reinsertion of NG tube, length of hospital stay and mortality.

### Data extraction and quality assessment

Three authors (K. A., V. P. and T. K.) extracted data from the included studies using predefined proformas. The quality of included studies was assessed using the ROBINS-I risk of bias in non-randomised studies of interventions^20^ and Cochrane Risk of Bias 2 tools^21^ to determine risk of bias in non-RCTs and RCTs, respectively.

### Statistical methods

A random-effects, pairwise meta-analysis was conducted in R (R Foundation for Statistical Computing, Vienna, Austria)^22^ with the metafor^23^ package. The Mantel–Haenszel method was employed, and the DerSimonian–Laird estimator for between-study variance. Weighted means were calculated by the generic inverse variance method. Baseline differences were compared with a random-effects, pairwise meta-analysis; continuous baseline variables were reported as weighted means. Odds ratios were presented for dichotomous variables, and mean differences (MD) for continuous variables with 95 per cent confidence intervals. Statistical heterogeneity was indicated by the *I*^2^ values whereby a threshold of 50 and 75 per cent were indicative of moderate and substantial heterogeneity, respectively. Publication bias was assessed by visual inspection of funnel plots. A sensitivity analysis was performed for primary outcomes after removal of the single RCT.

## Results

### Study and patient characteristics

**Fig. 1:**
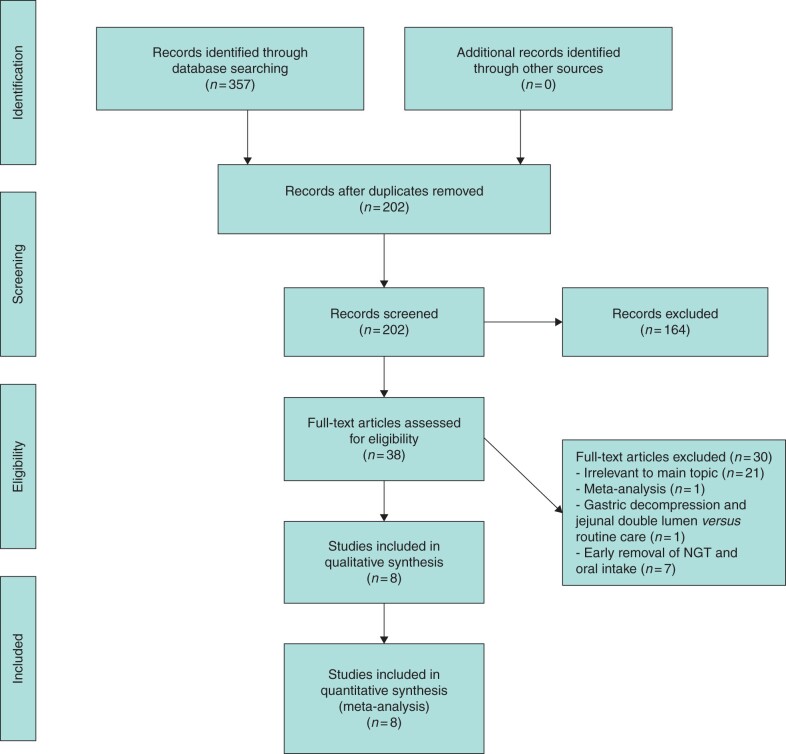
Preferred Reporting Items for Systematic Reviews and Meta-analyses (PRISMA) flow chart NGT, nasogastric tube.

Eight studies were included in the meta-analysis (*Fig. 1*), with a total of 1301 patients enrolled, of whom 668 had routine postoperative NGD. The study population’s baseline characteristics are summarised in *Table 1*. The male-to-female ratio was 660 : 640 (approximately 1 : 1). Studies were published between March 2011 and July 2020, and conducted in Norway^13^, France^15^^,^^29^, Korea^24^^,^^28^ and the United States^25–27^. One study was an RCT^15^, six were prospective comparative studies^13^^,^^25–29^ and one was a retrospective study^24^. The study characteristics are summarized in *Table 2*. A total of 92.5 per cent of patients underwent PD and 7.5 per cent underwent either a distal or a total pancreatectomy. Amongst those patients who underwent PD, 50.1 per cent underwent a classic PD, and 49.9 per cent a pylorus-preserving PD.

**Table 1:** Baseline characteristics of participants included in studies

Study	Study population	Sex M/F	Age (year) mean ± SD	Malignant/ benign indication	Preoperative DM	Preoperative BMI	Whipple’s/ PPPD	Blood loss (ml) mean ± SD
	NGD*	NGD	NGD	NGD	NGD	NGD	NGD	NGD
	No NGD†	No NGD	No NGD	No NGD	No NGD	No NGD	No NGD	No NGD
Kleive^13^		31/14	69.4 ± 6.8		10/35	25.4 ± 4.4	16/29	
	45/156	71/85	66.4 ± 10.1	N/A	26/130	24.3 ± 3.6	47/109	N/A
Bergeat^15^		38/21	62.8 ± 2.05	48/11	9/50	23.77 ± 0.99	59/0	203.95 ± 54.2
	59/52	31/21	63.82 ± 2.21	42/10	12/40	23.94 ± 1.32	52/0	206.76 ± 88.56
Choi^24^		9/9	61.22 ± 11.63		3/15		15/1	922.2 ± 357.37
	18/23	14/9	62.61 ± 10.01	N/A	3/20	N/A	16/6	1178.3 ± 506.28
Fisher^25^		24/26	64				1/30	
	50/50	20/30	62	N/A	N/A	N/A	1/32	N/A
Roland^26^		66/90	64.4 ± 10.18	117/39			113^‡^	
	156/75	32/43	62.6 ± 10.64	52/23	N/A	N/A	56^‡^	N/A
Kunstman^27^		64/61	63.15 ± 11.06	92/33	25/100		19/106	612.3 ± N/A
	125/125	57/68	63.68 ± 13.97	94/31	30/90	N/A	66/59	504.6 ± N/A
Park^28^		52/64	64.18 ± 10.58	95/21	25/91	23.3 ± 3.9	0/116	838.53 ± 509.67
	116/112	64/48	61.84 ± 9.25	88/24	23/89	22.7 ± 3.4	0/112	993.71 ± 484.55
Gaignard^29^		62/37	66.91 ± 2.7	77/22	18/81	24.02 ± 0.74	99/0	
	99/40	25/15	67.02 ± 3.29	25/15	6/34	24.17 ± 1.34	40/0	N/A
Overall§		346/322	64.80 (95% c.i. 62.74,66.93)	370/93	90/371	23.74 (95% c.i. 23.20,24.30)	322/282	539.39 (95% c.i. 179.89,1617.29)
	668/633	314/319	64.19 (95% c.i. 62.65,65.76)	238/727	100/403	23.81 (95% c.i. 23.25,24.38)	278/318	622.7 (95% c.i. 205.19,1890.16)
*P*-value¶		0.51	0.44	0.19	0.90	0.51	0.60	0.28

*Nasogastric decompression (NGD) via tube gastrostomy in Park study.

†The no NGD group in Kunstman study had nine of 125 patients who had a nasogastric tube postoperatively. ^‡^Roland *et al*. reported the number of pancreaticoduodenectomy, including Whipple’s and PPPD, collectively.

§Continuous variables are reported as weighted means.

¶*P*-values comparing pooled values between NGD and no NGD groups. N/A, not available; DM, diabetes mellitus; PPPD, pylorus-preserving pancreaticoduodenectomy.

**Table 2:** Study characteristics of included studies

Study characteristics	Country	Period of patient inclusion	Study design	Comparison groups	Selection to NGD *versus* no NGD based on	Inclusion and exclusion criteria
Kleive^13^	Norway	2 years (2015–2016)	Prospective observational	NGD *versus* no NGD reinsertion	NGT was removed immediately postoperatively in all patients and reinserted if indicated	Inclusion: All patients who underwent PD
						Exclusion: Other types of pancreatic resections
Bergeat^15^	France	2.6 years (January 2016–August 2018)	RCT	NGD *versus* no NGD	Randomized	Inclusion: All patients aged between 18 and 75 years requiring PD for benign or malignant biliopancreatic confluence lesions
						Exclusion: Previous gastric/ oesophageal surgery Severe co-morbidities Chronic respiratory disease Heart failure Pregnancy or nursing mothers Patients under guardianship
Gaignard^29^	France	2 years (2014–2015)	Prospective, comparative	NGD *versus* no NGD	Two cohorts: before May 2015, all patients had routine NGD; after May 2015, all patients had NGT immediately removed postoperatively	Inclusion: All patients who underwent PD
						Exclusion: N/A
Choi^24^	Korea	3 years (July 2004–May 2007)	Retrospective	NGD *versus* no NGD	N/A	Inclusion: All patients who underwent PD
						Exclusion: N/A
Park^28^	Korea	5 years (2009–2014)	Prospective, comparative	NGD *versus* no NGD	Two cohorts: before June 2012, all patients had routine NGD; after July 2012, all patients had NGT immediately removed postoperatively	Inclusion: Patients who underwent PPPD
						Exclusion: N/A
Fisher^25^	USA	2.75 year (January 2008–September 2010)	Prospective, comparative	NGD *versus* no NGD	Two cohorts: first 50 patients had routine NGD; second 50 patients had NGT immediately removed in operating room	Inclusion: 100 consecutive patients who underwent PD or DP
						Exclusion: Other types of pancreatic resections
Roland^26^	USA	13.5 years (1997 –May 2011)	Prospective, comparative	NGD *versus* no NGD	Two cohorts: before May 2006, all patients had routine NGD; after May 2011, all patients had NGT removed in operating room	Inclusion: All patients aged above 14 years and who underwent pancreatic resections
						Exclusion: N/A
Kunstman^27^	USA	8.5 years (July 2003–February 2012)	Prospective, comparative	Routine NGD *versus* selective NGD	Two cohorts: first 125 patients had routine NGD; second 125 patients had NGD only in selective indications	Inclusion: Patients undergoing PD
						Exclusion: N/A

NGD, nasogastric decompression; NGT, nasogastric tube; PD, pancreaticoduodenectomy; N/A, not available; PPPD, pylorus-preserving pancreaticoduodenectomy.

### Quality assessment and risk of bias

Risk of bias assessment for the single RCT by using the Cochrane Risk of Bias 2 tool showed a low risk of bias. The remaining non-RCTs showed a low risk of bias for three studies and a moderate risk of bias for four studies (*Table S1*).

## Primary outcome measures

### Definition of DGE

Six studies^13^^,^^15^^,^^25^^,^^27–29^ used the ISGPS definition of DGE, whereas two studies defined DGE differently. Choi *et al*. defined DGE as gastric stasis requiring an NG tube for more than 10 days or where a regular diet was not tolerable on POD 14^24^. Roland *et al*. diagnosed DGE if an NG tube was reinserted because of nausea and vomiting for more than 7 days and not tolerating an oral diet or hydration by day 10 or inability to tolerate an oral diet prolonging hospital stay by more than 2 days^26^.

### DGE

**Fig. 2:**
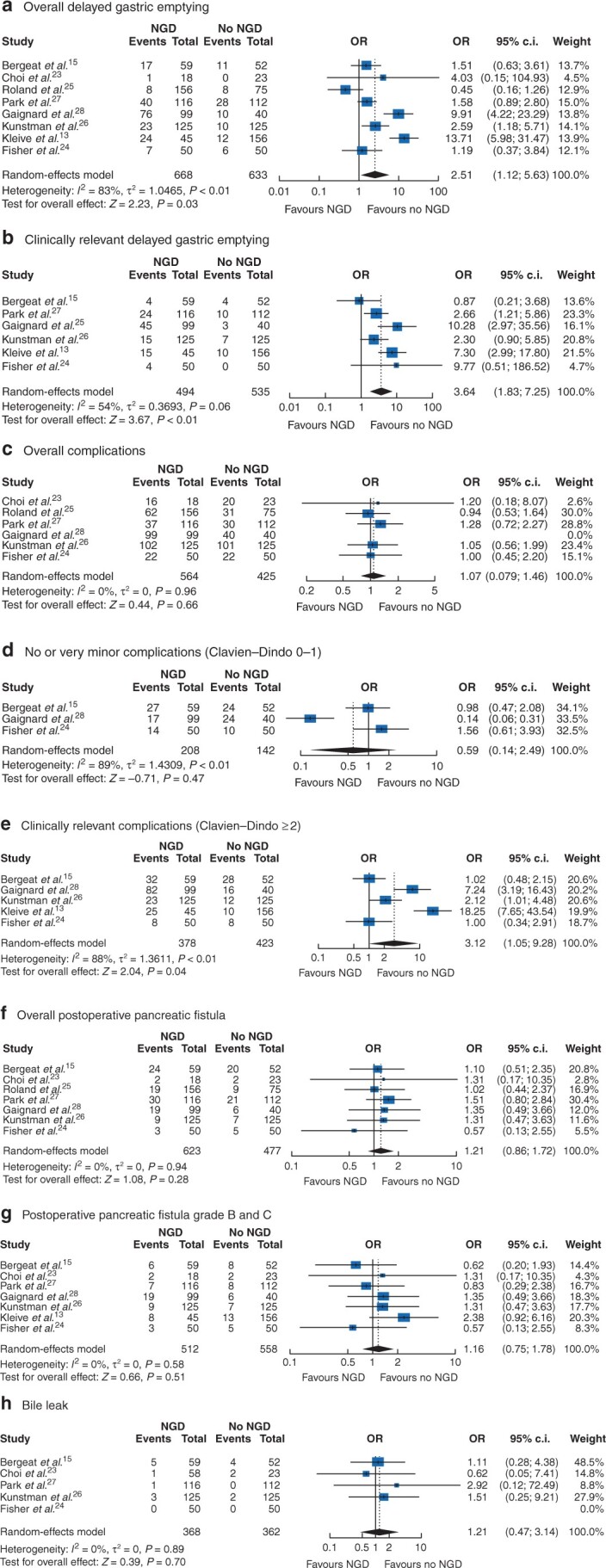
Primary outcomes The Mantel–Haenszel random-effects model was used for the meta-analysis of all outcomes. Odds ratio (OR) are shown with 95 per cent confidence intervals.

All studies were included in the analysis of overall DGE, with a total of 668 patients with routine NGD and 633 patients without^13^^,^^15^^,^^24–29^. NGD was associated with a higher rate of DGE: 29.3 per cent (196/668) *versus* 13.4 per cent (85/633) in those without NGD (odds ratio 2.51, 95 per cent c.i. 1.12 to 5.63, *I*^2^ = 83 per cent; *P* = 0.03) (*Fig. 2a*).

### DGE grades B and C (CR-DGE)

Six studies reported CR-DGE^13^^,^^15^^,^^25^^,^^27–29^, with a total of 494 patients in the NGD group and 535 patients in the no-NGD group. NGD was associated with a higher incidence of CR-DGE. The rate of CR-DGE in the NGD group was 16 per cent (107/668), and 5.3 per cent (34/663) in the no-NGD group (odds ratio 3.64, 95 per cent c.i. 1.83 to 7.25, *I*^2^ = 54 per cent; *P* < 0.01) (*Fig. 2b*).

A sensitivity analysis was performed after removal of the RCT, which showed lower rates of DGE (*P* = 0.03) and CR-DGE (*P* < 0.01) in the no NGD group (*Fig. S1*).

## Secondary outcome measures

### All complications

There were no significant differences in the overall complications (odds ratio 1.07, 95 per cent c.i. 0.79 to 1.46, *I*^2^ = 0 per cent; *P* = 0.66) (*Fig. 2c*). Similarly, there were no significant differences between the two groups in Clavien–Dindo grade 0–I complications (odds ratio 0.59, 95 per cent c.i. 0.14 to 2.49, *I*^2^ = 89 per cent; *P* = 0.47) (*Fig. 2d*). Clavien–Dindo grade II or higher complications occurred more frequently with NGD (odds ratio 3.12, 95 per cent c.i. 1.05 to 9.28, *I*^2^ = 88 per cent; *P* = 0.04) (*Fig. 2e*).

### Postoperative pancreatic fistula and bile leak

POPF was defined in four studies^24^^,^^25^^,^^27^^,^^28^ according to the ISGPF 2005 classification^1^, whereas three studies^13^^,^^15^^,^^29^ used the updated classification (ISGPF 2016)^19^.

There were no significant differences in overall POPF (odds ratio 1.21, 95 per cent c.i. 0.86 to 1.72, *I*^2^ = 0 per cent; *P* = 0.28) or clinically relevant POPF (grades B and C) (odds ratio 1.16, 95 per cent c.i. 0.75 to 1.78, *I*^2^ = 0 per cent; *P* = 0.51) (*Fig. 2f, g*). Similarly, there were no significant differences in rates of bile leak (odds ratio 1.21, 95 per cent c.i. 0.47 to 3.14, *I*^2^ = 0 per cent; *P* = 0.70) (*Fig. 2h*).

### Pulmonary complications

**Fig. 3:**
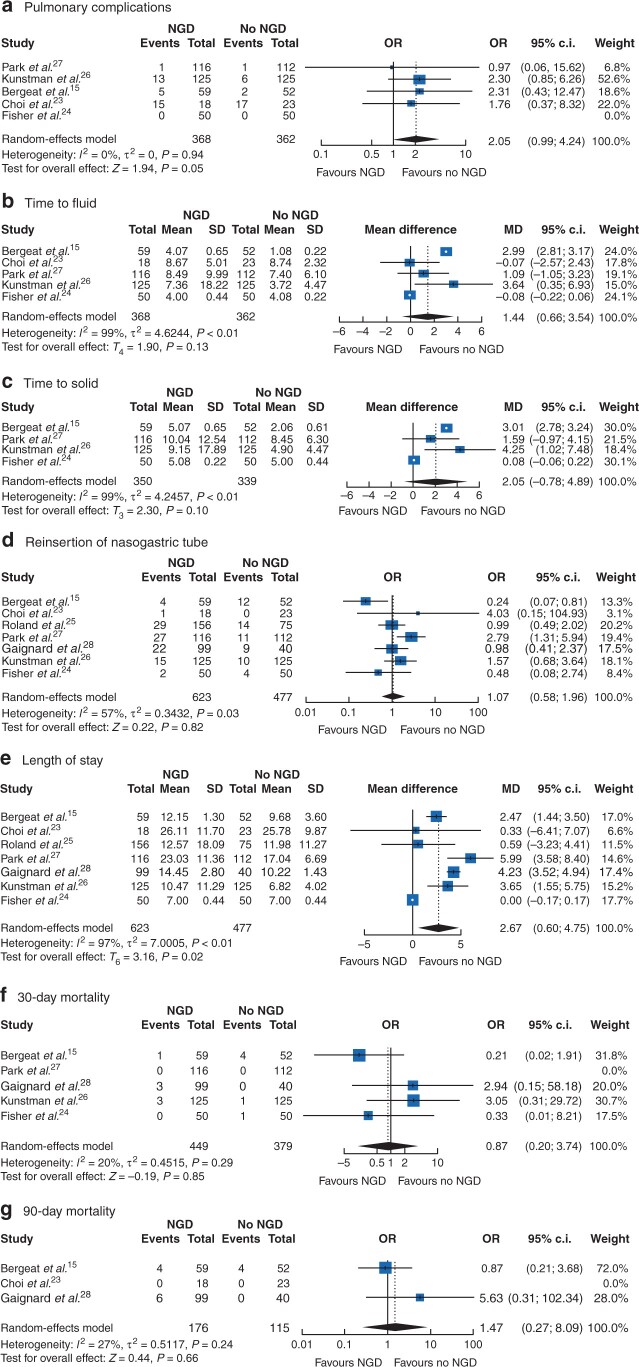
Secondary outcomes The Mantel–Haenszel random-effects model was used for the meta-analysis of all outcomes. Odds ratio (OR) are shown with 95 per cent confidence intervals.

There were no significant differences in pulmonary complications (odds ratio 2.05, 95 per cent c.i. 0.99 to 4.24, *I*^2^ = 0 per cent; *P* = 0.05) (*Fig. 3a*).

### Time to oral intake

There were no significant differences in time to first oral fluid (MD 1.44, 95 per cent c.i. −0.66 to 3.54, *I*^2^ = 99 per cent; *P* = 0.13) or solid intake (MD 2.05, 95 per cent c.i. −0.78 to 4.89, *I*^2^ = 99 per cent; *P* = 0.10) (*Fig. 3b, c*).

### Reinsertion of NG tube

The rate of reinsertion of an NG tube after removal in the NGD group was 16 per cent, whereas 12.5 per cent of patients required NG tube reinsertion in the no-NGD group (odds ratio 0.82, 95 per cent c.i. 0.58 to 1.96, *I*^2^ = 57 per cent; *P* = 0.82) (*Fig. 3d*).

### Length of hospital stay

The mean length of hospital stay with NGD was 5.40 ± 6.03, whereas without NGD, the mean length of hospital stay was 5.00 ± 3.82 (MD 2.67, 95 per cent c.i. 0.60 to 4.75, *I*^2^ = 97 per cent; *P* = 0.02) (*Fig. 3e*).

### Mortality

There were no significant differences in 30-day (odds ratio 0.87, 95 per cent c.i. 0.2 to 3.74, *I*^2^ = 20 per cent; *P* = 0.85) or 90-day mortality (odds ratio 1.47, 95 per cent c.i. 0.27 to 8.09, *I*^2^ = 27 per cent; *P* = 0.66) between the two groups (*Fig. 3f, g*).

## Publication bias

Funnel plots for publication bias are summarized in *Figs S2–S7*. There was no evidence of publication bias in overall complications, CR-DGE, POPF or mortality outcomes. There was publication bias in Clavien–Dindo grade 0–I and Clavien–Dindo grade II or higher complications, as well as in overall DGE, length of hospital stay and time to first oral fluid and solid intake.

## Discussion

The present systematic review and meta-analysis assessed the impact of routine NGD after PD. Results showed that routine NGD was associated with higher rates of DGE, CR-DGE, increased Clavien–Dindo grade II or higher complications, increased pulmonary complications and a longer hospital stay.

Despite the declining practice of NGD after major abdominal surgeries, some ambiguity remains with regard to use of routine NGD after PD^5^^,^^8^. This is largely due to a perceived increased risk of complications unique to PD such as DGE, POPF or biliary leakage. NGD is thought to decompress the stomach and reduce tension on the gastroenteric anastomosis, potentially leading to a decreased risk of anastomotic leaks and overall morbidity associated with PD. The ERAS 2019 recommendations to remove NG tubes before reversal of anaesthesia in PD have not been adopted by most surgeons, as the impact of removal of NG tubes on POPF and DGE rates was not clear^13^^,^^16^^,^^29^^,^^30^. Moreover, the rate of reinsertion of NG tubes was not known. The majority of the studies on which the ERAS recommendations were based were retrospective in nature, included small sample sizes and were single-centre^13^^,^^16^^,^^29^^,^^30^.

DGE occurs in 10 to 45 per cent of patients after PD^31–34^. Risk factors for DGE include POPF, postoperative complications and potentially reconstruction technique^35–37^. The pathophysiology of DGE after PD remains poorly understood. Gastric accommodation, gastroduodenal pressure gradients and antro-pyloric coordination may be impaired after a classic Whipple’s, and these factors likely play an important role in DGE^38–40^. It remains unclear if NGD is favourable for DGE in the context of altered anatomy and motility patterns of the stomach after PD. Post-PD DGE remains complex and is likely multifactorial. However, evidence from this review suggests routine NGD may not be as beneficial as has been commonly thought. The fact that complications remained similar between groups, in particular POPF, and yet DGE rates were higher in those receiving routine NGD, adds confidence to the finding that DGE may be associated with routine NGD.

All included studies in this systematic review and meta-analysis were consistent in recommending against routine NGD after PD^13^^,^^15^^,^^24–29^. Kunstman *et al*. reported a lower incidence of DGE in those without routine NGD^27^. The incidence of CR-DGE was also lower in those without routine NGD^29^. Two studies also reported significantly higher rates of postoperative Clavien–Dindo grade II or higher complications with routine NGD^13^^,^^29^. Several studies reported shorter length of stay^25^^,^^27^^,^^29^. The only randomized study included in this meta-analysis^15^ reported no significant difference between the two groups in Clavien–Dindo grade II or higher complications (*P* > 0.99), pulmonary complications (*P* = 0.44), DGE (*P* > 0.99) or length of stay (*P* = 0.14)^15^. The critical limitation of this single-centre RCT that included 125 patients was it was underpowered, with over 1200 patients needed in each arm to detect a 5 per cent difference in Clavien–Dindo grade II or higher complications. Therefore, taking these findings into account in the context of the entire literature that was synthesized in this review, the evidence recommends against routine NGD after PD. Additionally, this RCT was assessing superiority, and not non-inferiority^15^. Furthermore, the RCT was unable to evaluate quality of life or patient-related outcome measures^15^. Only one study in the present meta-analysis assessed patient discomfort. Future research should include quality of life metrics and patient perspectives and preferences.

A further search of ClinicalTrials.gov, The Netherlands Trial Register and Cochrane Library databases found no further ongoing RCTs on this topic. This may be due to impracticalities of adequately powering such studies of NGD after PD, given the PD caseloads within institutions to recruit for such studies. It may be informative to capture global variations in practice through multinational collaborative studies, such as those proposed in other specialties^41^.

There are several limitations to the present review. First, only one RCT was available for inclusion. It is therefore difficult to account for confounding factors in this analysis. Second, study cohorts were sometimes heterogenous, including distal and total pancreatectomies, although these accounted for less than 10 per cent of the overall cohort. Additionally, it is difficult to assess causality due to the preponderance of retrospective and non-randomized studies in the meta-analysis. Between-study heterogeneities were mitigated for by using a random-effects meta-analysis. Primary and secondary outcomes also varied amongst included studies. Despite this, however, generally balanced distribution of pylorus-preserving PD and classic Whipple’s in the included studies further increases the external validity of these findings.

This systematic review corroborates the recent IPOD-trial and the 2013 ERAS guidelines in recommending against routine NGD after PD. NGD may, in fact, increase DGE rates^13–15^. Further reductions in DGE rates likely require an improved understanding of its pathophysiology to inform novel, mechanistically guided management strategies. Future studies investigating gastric emptying and gastric physiology, potentially through novel non-invasive technologies, may further advance the understanding of the pathophysiology of DGE after PD. Newer tools such as body surface gastric mapping may also reveal novel insights into gastric dysrhythmias that may be implicated in DGE after PD^42^^,^^43^. Given the implication that routine NGD is not a suitable prophylactic measure for DGE, these other avenues require investigation. Further optimization of the surgical technique^44^ and a better understanding of the underlying pathophysiology may guide management.

## Funding

This research did not receive any specific grant from funding agencies in the public, commercial or not-for-profit sectors.

*Disclosure*. The authors declare no conflict of interest.

## Supplementary material

Supplementary material is available at *BJS Open* online.

## Supplementary Material

zrab111_Supplementary_Data
