# Supplementary material for: Impact of routine nasogastric decompression versus no nasogastric decompression after pancreaticoduodenectomy on perioperative outcomes: meta-analysis
Source: BJS Open. 2021 Dec 21;5(6):zrab111. doi: 10.1093/bjsopen/zrab111 (PMC8691053; doi:10.1093/bjsopen/zrab111)
Supplement: zrab111_Supplementary_Data [file zrab111_supplementary_data.zip › Supplementary_material.docx]

**Supplementary material**

**Table S1: Risk of bias assessment**

| **Risk of bias for RCT** | **Randomisation** | **Deviation from intended interventions** | **Missing outcome data** | **Measurement of outcome** | **Selection of outcome reporting** | **Overall** |
| --- | --- | --- | --- | --- | --- | --- |
| Bergeat et al ^15^ | Low | Low | Low | Low | Low | Low |
| **ROBINS-1** | **Confounding** | **Selection** | **Deviation from intervention** | **Missing data** | **Outcome’s measurement** | **Reporting** |
| Park et al ^25^ | Low | Low | Medium | Low | Medium | Medium |
| Gaignard et al ^23^ | Low | Medium | Medium | Low | Low | Medium |
| Kunstman et al ^28^ | Low | Low | Low | Low | Low | Low |
| Kleive et al ^13^ | Medium | Medium | Low | Low | Low | Medium |
| Fisher et al ^26^ | Low | Low | Low | Low | Low | Low |
| Roland et al ^27^ | Low | Low | Medium | Low | Medium | Medium |
| Choi et al ^24^ | Low | Low | Low | Low | Low | Low |

**Figure S1.** A Forest plot of sensitivity analysis of delayed gastric emptying and clinically relevant delayed gastric emptying after removal of the randomised control trial.

**
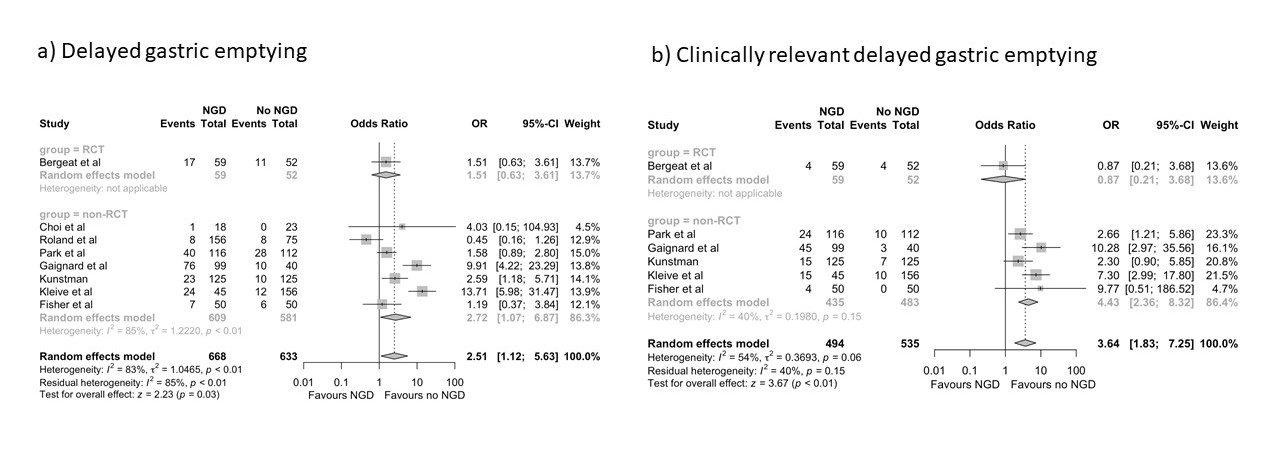
**

NGD: nasogastric decompression

**Publication bias:**

**Figure S2.** Funnel Plot of Standard Error by Odds ratio, for overall delayed gastric emptying **
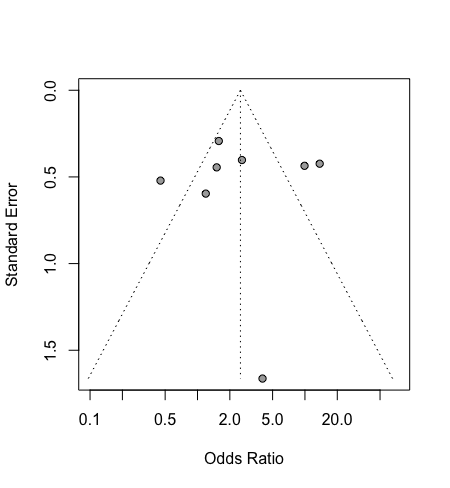
**

**Figure S3.** Funnel Plot of Standard Error by Odds ratio, for clinically relevant delayed gastric emptying

**
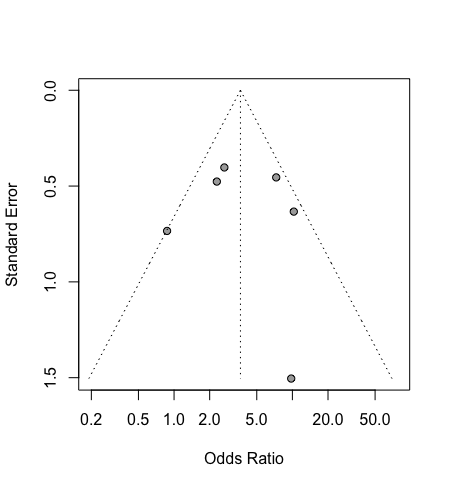
**

**Figure S4.** Funnel Plot of Standard Error by Odds ratio, for pancreatic fistula grade B and C

**
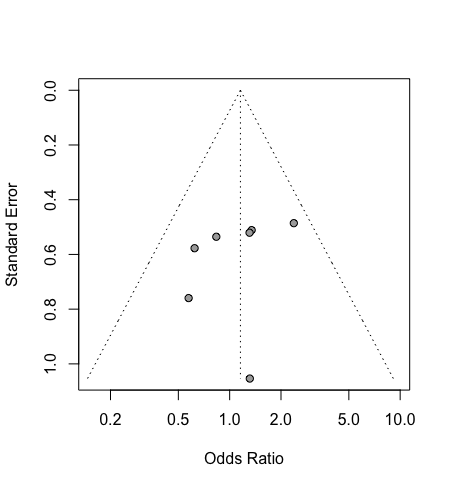
**

**Figure S5.** Funnel Plot of Standard Error by Standard difference in means, for time to oral fluid in take

**
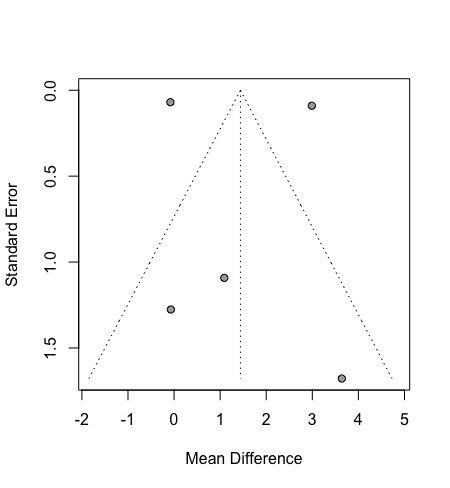
**

**Figure S6.** Funnel Plot of Standard Error by Standard difference in means, for time to solid oral intake.

**
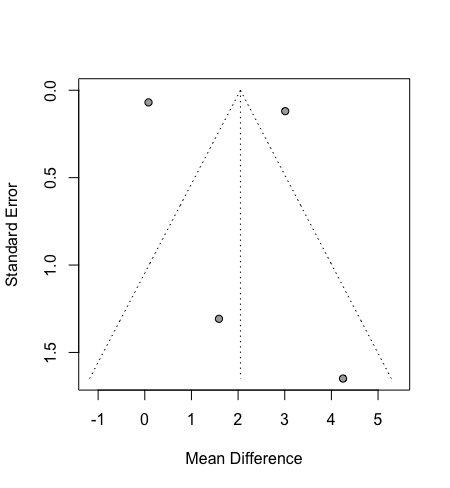
**

**Figure S7.** Funnel Plot of Standard Error by Standard difference in means, for length of stay

**
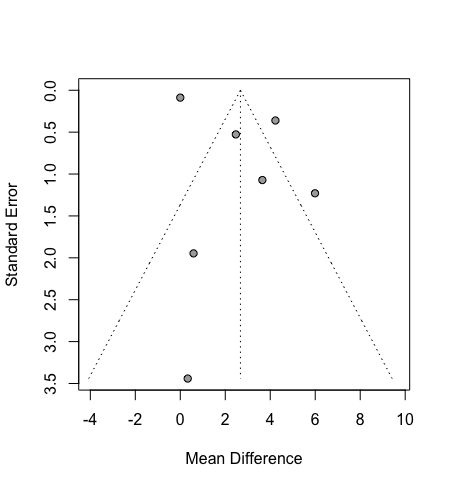
**
